# Structural Characterization, Antimicrobial, Antibiofilm, Antioxidant, Anticancer and Acute Toxicity Properties of N-(2-hydroxyphenyl)-2-phenazinamine From *Nocardiopsis exhalans* (KP149558)

**DOI:** 10.3389/fcimb.2022.794338

**Published:** 2022-05-19

**Authors:** Vaikundamoorthy Ramalingam, Rajendran Rajaram, Govindaraju Archunan, Parasuraman Padmanabhan, Balázs Gulyás

**Affiliations:** ^1^ Centre for Natural Products and Traditional Knowledge, Indian Institute of Chemical Technology, Hyderabad, India; ^2^ DNA Barcoding and Marine Genomics Lab, Department of Marine Science, Bharathidasan University, Tiruchirappalli, India; ^3^ Department of Animal Science, Bharathidasan University Tiruchirappalli, Tamil Nadu, India; ^4^ Dean of Research, Marudupandiyar College, Thanjavur, India; ^5^ Lee Kong Chian School of Medicine, Nanyang Technological University, Singapore, Singapore; ^6^ Cognitive Neuroimaging Centre, Nanyang Technological University, Nanyang Technological University, Singapore, Singapore; ^7^ Imaging Probe Development Platform (IPDP), Nanyang Technological University, Singapore, Singapore

**Keywords:** *Nocardiopsis exhalans*, antimicrobial, antibiofilm, antioxidant, anticancer, acute toxicity

## Abstract

The present study aimed to isolate and identify potential drugs from marine actinomycete *Nocardiopsis exhalans* and screen them for biomedical applications. The cell-free culture of *N.* exhalans was extracted with ethyl acetate and the solvent extract showed six fractions in thin-layer chromatography. The fractions were subjected to column chromatography for purification and evaluated for activity against human clinical pathogens. Fraction 4 showed significant activity and was identified as N-(2-hydroxyphenyl)-2-phenazinamine (NHP) using spectral analyses. Further, NHP showed excellent biofilm inhibitory activity against human clinical pathogens *Escherichia coli*, *Pseudomonas aeruginosa*, and *Staphylococcus aureus*. The *in vitro* antioxidant activity confirmed that NHP is scavenging the oxidative stress-enhancing molecules. The anti-proliferative activity of NHP against human breast cancer cells showed significant activity at 300 µg/ml and less cytotoxic activity against normal cells. Additionally, the toxicity assessment against zebrafish revealed that NHP does not cause any toxicity in the important organs. The results highlight *N. exhalans* as a promising candidate for the development of antibiotics with potential therapeutic applications.

## 1 Introduction

Antimicrobial resistance (AMR) considerably has a great impact on human health and is predicted to increase the global mortality and economic burden (Manesh and Varghese, 2021). Among the diverse regulators of AMR, poor cleanliness and infection control, the consumption of weak or excluded medicines, second quality of antibiotics, and overuse of clinically relevant drugs for agriculture and veterinary systems are contributing factors to the emergence of AMR (Aslam et al., 2018; Lewnard et al., 2020). Multidisciplinary and comprehensive efforts are necessary to control the rise of resistance by deciphering the emergence of microorganisms, mechanism of action, and role of antibiotics (Knight et al., 2021). Excessive usage of antibiotics is associated with severe side effects, so researchers focus on novel antibiotics without toxicity. Hence, natural products from marine microbes have always attracted them for the development of antibiotics against AMR.

Microbial diversity aids to build a new generation of unique structures, enabling a beneficial source for novel pharmaceuticals and other high-value products (Abdel-Razek et al., 2020). An ample quantity of valuable bioactive constituents and drugs has been identified from terrestrial microorganisms (Shrestha et al., 2021); however, studies in this area have reduced due to the feeling that this resource has been fully harvested. Therefore, researchers focus on the marine-derived antibiotics to develop new therapeutic applications for the modern world. Apparently, microorganisms are extensively scattered all over the marine environment including intertidal zones, water, animals, and macroalgae and in sediment samples (Troussellier et al., 2017; Palladino et al., 2021). Among these microorganisms, marine actinomycetes have received more attention from the researchers due to their immense potential to produce secondary metabolites.

Recently, culture-dependent and metagenomic studies suggest that actinomycetes originally originated from the marine environment (Rego et al., 2019). Most of the marine actinomycetes grow in mediums prepared using sea water, and this is an exceptional adaptive feature of marine actinomycetes (Subramani and Sipkema, 2019). These kinds of rare actinomycetes are the origin of a fascinating study to isolate novel actinomycetes and also the key for unique antibiotics and metabolites with biomedical applications (Ding et al., 2019). Despite this key source, comparatively limited studies have been reported on marine actinomycetes and very few studies have been reported for chemical fingerprinting (Jose and Jha, 2016). Most of the chemical constituents reported for novel medicinal applications have the capability to be commercialized as therapeutics (Kamjam et al., 2017). Hence, the present study focuses on the isolation of *Nocardiopsis exhalans* from the Scleractinia coral *Acropora formosa* and determination of its bacterial growth inhibitory activity. The potential fraction was purified and identified as N-(2-hydroxyphenyl)-2-phenazinamine (NHP) using modern characterization techniques. Moreover, the isolated compound was assessed for its antibiofilm efficiency on clinical pathogens, antioxidant activity, anticancer activity against breast cancer, and acute toxicity against *Danio rerio*.

## 2 Materials and Methods

### 2.1 Isolation and Identification of Actinomycetes

The coral surface mucus layer was collected from the *A. formosa* collected from Keelakarai, Gulf of Mannar, southeast coast of India. The mucus samples were collected and kept in 1 ml of sterilized sea water and transferred to the laboratory. Actinomycetes were isolated using the starch casein agar (HiMedia Laboratories, Mumbai, India) plating technique as described earlier (Nithyanand et al., 2011). A total of 21 actinomycetes were isolated and evaluated for growth inhibitory activity against human clinical pathogens (**Table S1**) using the streak plate method. Among the 21 actinomycetes, the isolate A.f – 4 had potential antimicrobial activity in primary screening and was chosen for mass culture for the production of bioactive constituents (**Table S2**). The single colony of A.f – 4 strain was inoculated in Yeast Malt Agar (ISP2) medium, and the pellet was collected after spin at 6,000 *g* for 10 min. Genomic DNA was isolated, and the 16S rRNA gene was amplified from actinomycetes according to the previous method (Babu et al., 2009) and identified as *N. exhalans* (KP149558) as well as phylogenetic analysis (**Figure S1**).

### 2.2 Production of Bioactive Compounds

The production of bioactive compounds from the marine actinomycete *N. exhalans* was carried out as reported previously (Shobha and Onkarappa, 2011). In brief, the pure culture of *N. exhalans* was grown in ISP2 medium for a week at room temperature in shaking condition. The culture was centrifuged at 10,000 *g* for 20 min, and a membrane filter (0.2 μm pore size) was used to separate the mycelial biomass. An equal volume of ethyl acetate was mixed with a cell-free extract and was homogenized for 1 h. The solvent was centrifuged at 6,000 *g* for 20 min, and the debris was discarded (Nithyanand et al., 2009). The cell-free extract was investigated for bacterial growth inhibitory efficiency against human clinical pathogens.

### 2.3 Characterization

The crude *N. exhalans* exhibiting growth inhibitory activity was eluted with a methanol: chloroform (6:4) solvent system. The fractions were collected, and the solvent was evaporated using a rotary evaporator. The fractions (2 g) were subjected to silica gel column chromatography (100–200 mesh size) for purification, and the methanol and chloroform (6:4) solvent system was used for elution. The active fraction (1 mg) was mixed with KBr to obtain a pellet, and the Fourier-transform infrared spectroscopy (FTIR) spectra (SAM-AV spectrum, model – Spectrum RXI) were recorded. The active fraction (5 mg) was further analyzed using Gas chromatography–mass spectrometry (GC-MS) (Shimadzu QP-2010 Plus) and Nuclear Magnetic Resonance (NMR) (Bruker Avance III) as described earlier (Ramalingam and Rajaram, 2016).

### 2.4 Antibiofilm Activity

#### 2.4.1 Culture of Bacteria

Human clinical pathogens such as *Escherichia coli* (MH701895), *Pseudomonas aeruginosa* (MH703431), and *Staphylococcus aureus* were collected from the Government Hospital, Tiruchirappalli, Tamil Nadu, India. Pure colonies were inoculated in a Luria-Bertani (LB) broth (HiMedia) for 24h and stored in glycerol at -70°C. The liquid culture of bacteria (OD_600_=1) used for further analysis was grown at 37°C and orbital shaking at 150rpm in the LB broth.

#### 2.4.2 *In Vitro* Antibiofilm Activity

NHP was assessed for its primary antibiofilm activity against *E. coli*, *P. aeruginosa*, and *S. aureus* and was initially confirmed using the UV spectrometric method (Ramalingam et al., 2019). Briefly, the pure colony cultured in the LB broth for 24 h at 37°C and 200 μl (OD_600 nm_ = 1.0) of the culture was inoculated in a 96-well polystyrene plate with various amounts of NHP (0–200 µg/ml). After incubation for 24 h at 37°C, the biofilm inhibitory efficiency of NHP was determined using the crystal violet staining assay by recording the absorbance at 570 nm.

#### 2.4.3 Confocal Laser Scanning Microscopy Analysis

Further, the effect of NHP on the structure of the pathogens during biofilm formation was observed using Confocal Laser Scanning Microscopy (CLSM) analysis as described earlier (Vaikundamoorthy et al., 2018). The bacteria in the liquid (OD_600 nm_ = 1.0) were cultured on a coverslip (13 mm) kept overnight a 6-well plate as defined above and treated with NHP for 24 h. After incubation, the cells were washed three times with PBS and the cells were stained with 0.01% of acridine orange. The architecture of the biofilm formation in the control and NHP-treated pathogens was observed under CLSM (CLSM, LSM 710; Carl Zeiss, Jena, Germany).

### 2.5 Antioxidant Activity

The effect of NHP against oxidative stress-inducing molecules was studied using various *in vitro* antioxidant assays. The scavenging of DPPH (2,2-diphenyl-1-picryl-hydrazyl-hydrate) free radical, hydrogen peroxide (H_2_O_2_), and hydroxyl radical and the reducing ability of ferric chloride by NHP were assessed as mentioned earlier (Ramalingam and Rajaram, 2018). Butylhydroxytoluene (BHT) was used a positive control.

### 2.6 Anticancer Activity

#### 2.6.1 Cell Lines and Culture

The human breast cancer cell line (MCF7) and HBL 100 normal cell lines were procured from the National Centre for Cell Science, Pune, India. The cells were maintained in Dulbecco’s Modified Eagle Medium with 10% of fetal bovine serum and 100 units/ml of penicillin and streptomycin at 37°C in 5% CO_2_ and 95% relative humidity. Culture media were replaced at least twice a week.

#### 2.6.2 Cytotoxic Activity of N-(2-hydroxyphenyl)-2-phenazinamine

The *in vitro* cytotoxic activity of NHP was evaluated against MCF7 breast cancer cell lines and HBL 100 normal cell lines using the 3-(4,5-dimethylthiazol-2-yl)-2,5-diphenyltetrazolium bromide (MTT) assay (Mohan et al., 2020). Initially, 1×10^5^ cells were added in 96-well plates and cultured for 24 h for attachment. The old culture medium was changed with a medium comprising various concentrations of NHP (50–500 µg/ml). After incubation for 24 h, the cells were rinsed three times with a 1× PBS buffer and the MTT solution (5 mg/ml) was added. After being incubated at 37°C for 2 h, the formed formazan crystals were diluted in 1 ml of Dimethylsulfoxide (DMSO) and the absorbance was measured at 570 nm.

#### 2.6.3 Effect of N-(2-hydroxyphenyl)-2-phenazinamine on Apoptosis of MCF7 Cells

The apoptosis effect of NHP against MCF7 breast cancer cells was observed using 2’,7’-dichlorodihydrofluorescein diacetate (DCFH-DA) and the acridine orange/ethidium bromide (AO/EB) staining assay. In brief, the MCF7 cells (1 × 10^5^ cells) were cultured overnight in a 12-well plate and the cells were incubated with IC_50_ concentration of NHP (300 µg/ml). After 24 h incubation, the cells were rinsed three times with 1× PBS and stained with 10 µl of DCFH-DA staining for 30 min in dark conditions. On the other hand, the cells treated with NHP were rinsed thrice with 1× PBS and stained with AO/EB (50 μg/ml in 1× PBS) for 5 min. After staining, the cells were visualized using a fluorescence microscope at 485 nm excitation and 520 nm emission for DCFH-DA staining (Ramalingam et al., 2020a) and 480/430 nm for the AO/EB staining assay (Ramalingam et al., 2020b).

### 2.7 *In Vivo* Toxicity Studies

The toxic effect of NHP was studied on *D. rerio* by following the animal ethical guidelines of Bharathidasan University. The zebrafish was procured from the local fish market and grown under laboratory environments with 12 h of light, 12 h of darkness, and 28 ± 2°C during the treatment time. For *in vivo* toxicity, the adult fish (12 numbers) were stocked in a 10 L container and treated with 500 µg/L of NHP for 21 days and the fish treated with water alone was considered as control. The old water was replaced with fresh water every 24 h, and mortality was observed daily. After 21 days, the control and experimental fishes were sacrificed and the major organs were separated to determine the toxicity level of NHP on fish by observing the morphological parameters of major organs. All the organs were stained with hematoxylin and eosin, and the images were acquired using a confocal laser scanning microscope and processed using Zen 2011 software (Carl Zeiss).

### 2.8 Statistical Analysis

All the experiments were performed in triplicate and the statistical significance of differences in the measured mean values were well determined using the Student’s two-tailed t-test. A *p*-value <0.05 was considered significant.

## 3 Results

### 3.1 Antimicrobial Activity of Ethyl Acetate Extract

**Figure 1:**
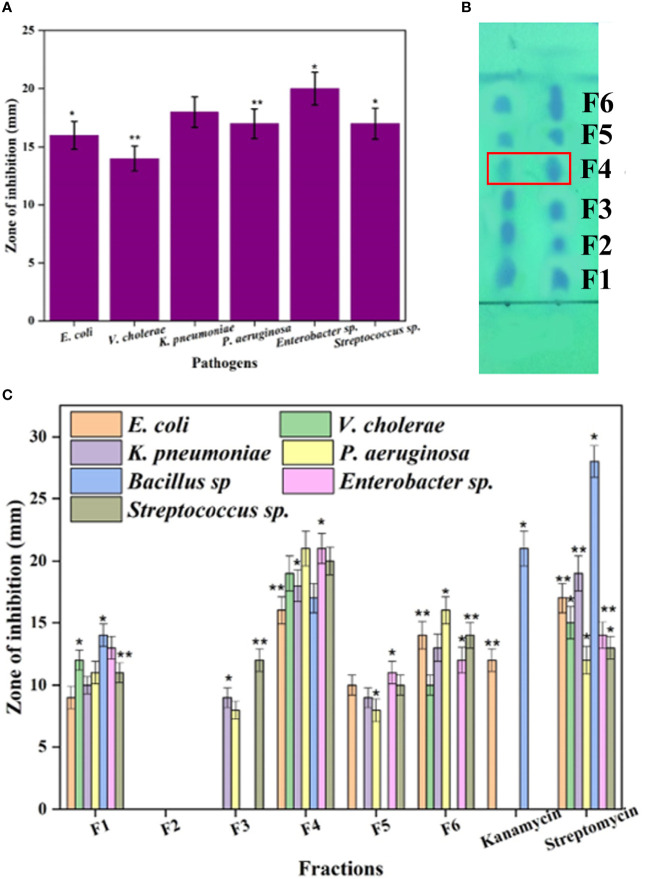
The antimicrobial activity of the ethyl acetate extract of N. exhalans **(A)** and TLC chromatogram of the ethyl acetate extract **(B)** (duplicate lanes). The spots were detected by exposing the TLC plate to UV light. The TLC-separated spots (F1–F6) were eluted and further purified by silica gel column chromatography using chloroform and methanol solvent as described in the results. Fractions 1–6 were tested for antimicrobial activity **(C)**. Results were taken in triplicates and expressed as mean ± standard deviation, and the significant differences were *p < 0.05 and **p < 0.01.

The present study was performed to search the novel bioactive compounds from marine actinomycetes *N. exhalans* isolated from the mucus of Scleractinia coral *A. formosa*. A total of 21 mucus-associated actinomycetes were isolated, and the potential antimicrobial activity-possessing strain was selected for mass culture. The selected actinomycetes *N. exhalans* showed significant antimicrobial activity in primary and secondary screening and was thus selected for mass culture for bioactive compound production. The selected actinomycete was cultured in the ISP2 medium for 7 days, and the fermentation was aborted. Then, the supernatant was collected after centrifugation at 10,000 *g* for 10 min. Likewise, in the present study, the ethyl acetate was used for the extraction of active compounds that were evaluated for bacterial growth inhibitory activity against human clinical pathogens. As shown in **Figure 1A**, the crude ethyl acetate extract of *N. exhalans* showed good growth inhibitory activity against clinical pathogens and the results confirmed that the extract of *N. exhalans* has potent bioactive constituents that suppress the growth of human pathogens.

### 3.2 Thin Layer Chromatography (TLC) Chromatogram and Antimicrobial Activity

The crude ethyl acetate extract showed significant antimicrobial activity against the tested pathogens (**Figure 1A**). To isolate active compounds from the crude ethyl acetate extract, a TLC chromatogram was performed on silica gel using a methanol–chloroform (6:4) solvent system (**Figure 1B**). Further, the TLC-separated spots (F1–F6) were subjected to column chromatography affording six fractions each with varying biological activity that were collected by eluting the column by increasing the polarity of solvent with chloroform–methanol from 10% to 40% of methanol. The six fractions were collected and evaluated for antimicrobial activity against human clinical pathogens. Among the six fractions, the F4 fraction showed excellent activity and the rest of them showed moderate activity (**Figure 1C**). Thus, the potent F4 fraction was purified by column chromatography (silica 100–200 mesh) and the column was eluted with the chloroform–methanol solvent system. The white amorphous powder was obtained and has been selected for further characterization.

### 3.3 Characterization of F4 Fraction

**Figure 2:**
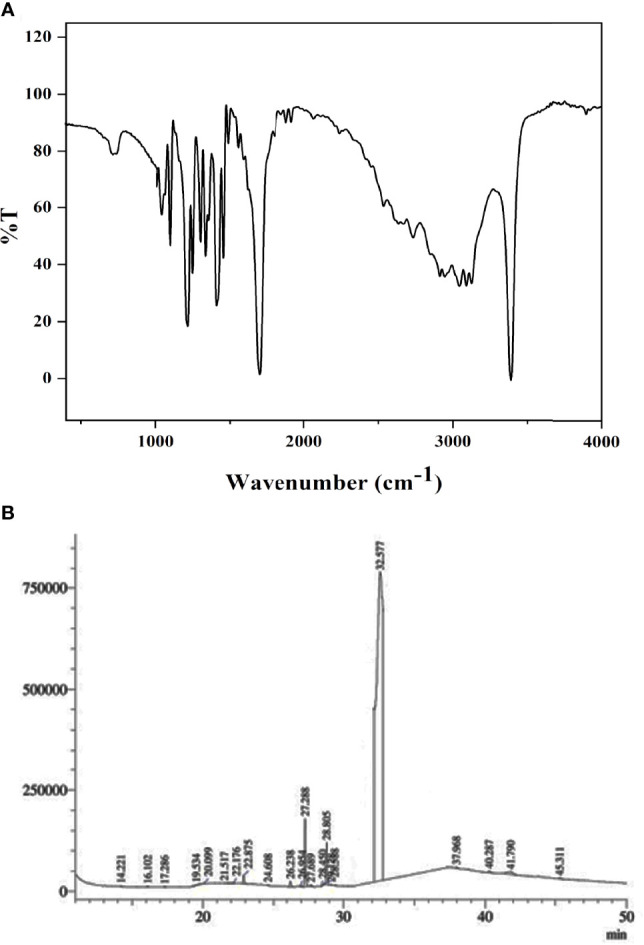
FTIR **(A)** and GC-MS **(B)** analysis of p`urified fraction 4 obtained from ethyl acetate extract of *N. exhalans*.

The isolated pure compound from *N. exhalans* was analyzed in FTIR, showing the presence of aromatic carbon at 1,680–1,100 cm^-1^ due to the carbon–carbon stretching, and the OH stretching was observed at 3,300–3,200 cm^-1^ (**Figure 2A**). The FTIR spectrum revealed that the effective groups observed in the compound could conceivably impact the bacterial inhibitory and antioxidant activities. The GC-MS analysis of the purified compound from *N. exhalans* showed 22 volatile compounds with different time intervals, and the highest peak was observed at 32.577 RT and identified as N-(2-hydroxyphenyl)-2-phenazinamine from mass spectrum analysis (**Figure 2B**).

**Figure 3:**
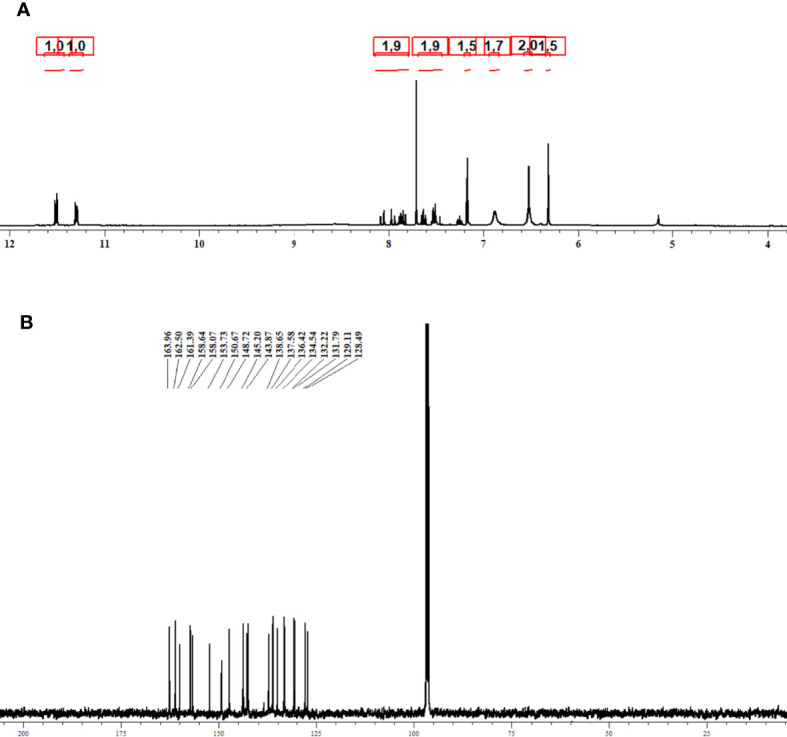
^1^H NMR **(A)** and ^13^C NMR **(B)** spectrum analysis of purified fraction 4 obtained from ethyl acetate extract of *N. exhalans*.

**Figure 4:**
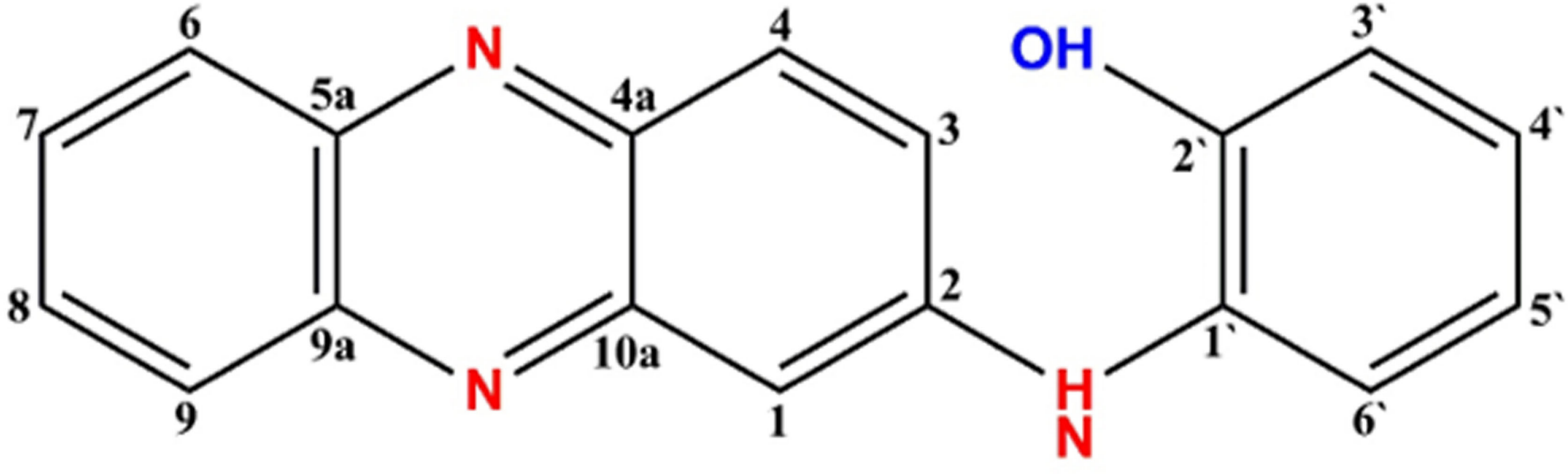
Structure of NHP and the molecular weight of NHP was found to be 287.25 m/z.

The purified compound isolated from actinomycetes strain *N. exhalans* was used for NMR analysis to interpret the structure of the compounds. The ^1^H NMR spectrum revealed that the presence of the aromatic hydrogens was observed between δ 8.516 and 6.205 (**Figure 3A**), and the ^13^C NMR spectrum revealed the aromatic carbons at 128–130 ppm (**Figure 3B**). The molecular weight of the compound was confirmed with mass data analysis, and the results showed that the molecular weight of NHP was 287.32 and found at 287.25 m/z (**Figure S2**). Taken together, the results confirmed that the purified compound derived from actinomycetes strain *N. exhalans* was N-(2-hydroxyphenyl)-2-phenazinamine (**Figure 4**).

### 3.4 Antibiofilm Activity

**Figure 5:**
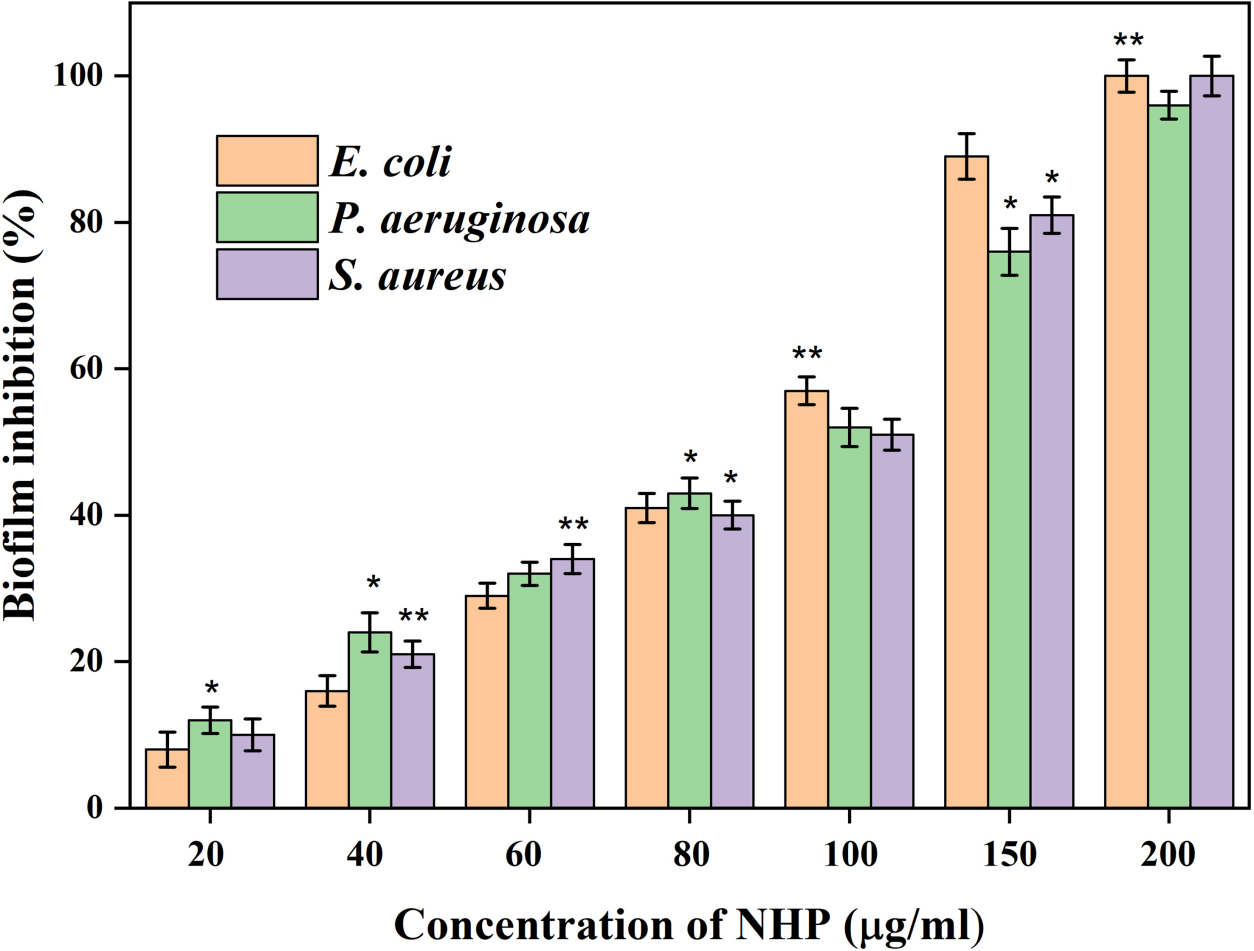
The biofilm inhibitory effect of different concentrations of NHP (0–200 μg/ml) on 24 h old mature biofilm measured using crystal violet staining assay. The results were taken in triplicates and expressed as mean ± standard deviation, and the significant differences were *p < 0.05 and **p < 0.01.

**Figure 6:**
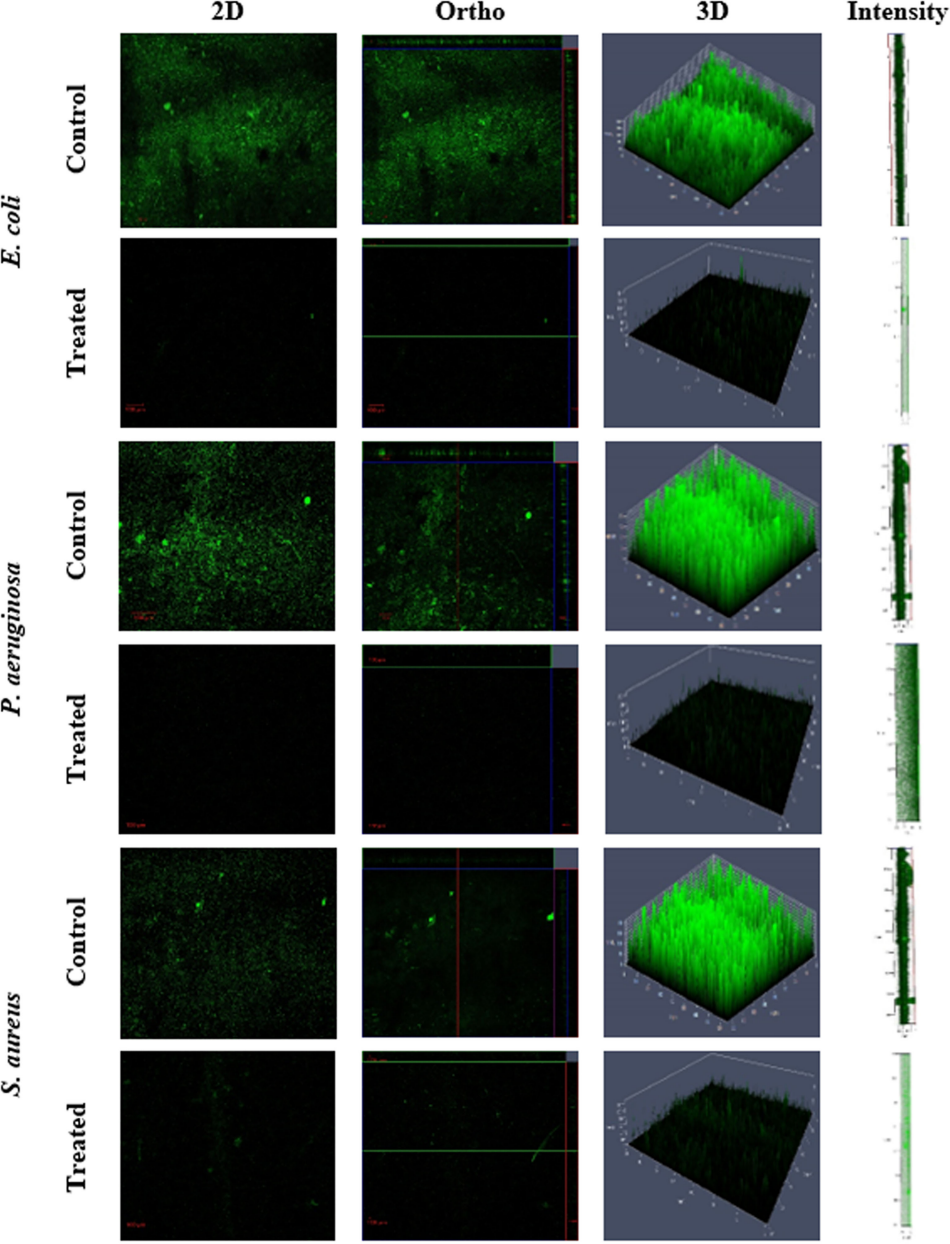
Confocal scanning laser microscopy images of biofilm formation on glass microscopic coverslips of control and NHP-treated (200 µg/ml) human clinical pathogens *E. coli*, *P. aeruginosa*, and *S. aureus*.

In the present study, the influence of NHP against the biofilm formation was initially assessed using the crystal violet staining assay and the outcomes are shown in **Figure 5**. It is obvious that increasing the NHP concentration (20–200 µg/ml) meritoriously inhibited the growth of biofilm-forming bacteria and approximately 100 µg/ml of NHP was calculated as biofilm inhibitory concentration against the tested clinical pathogens. Moreover, the higher concentration of NHP (200 µg/ml) inhibited the 100%, 98%, and 100% growth of *E. coli*, *P. aeruginosa*, and *S. aureus*, respectively. Further, NHP was assessed on the effect of the two-dimensional architecture of biofilm formation using CLSM analysis and the results showed that the control pathogenic bacteria have a good number of cell density and architecture. However, the NHP treatment exhibited the reduction in the cell density; the damage on the architecture of the bacteria and a lesser number of micro-colonies were observed in comparison with the control (**Figure 6**).

### 3.5 Antioxidant Activity

**Figure 7:**
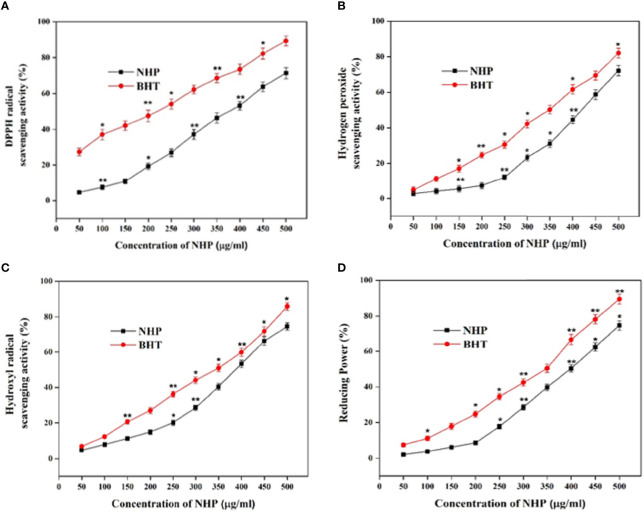
DPPH scavenging **(A)**, H_2_O_2_ **(B)**, hydroxyl radical scavenging **(C)**, and reducing power **(D)** activity of NHP obtained from ethyl acetate extract of *N. exhalans.* The results were taken in triplicates and expressed as mean ± standard deviation, and the significant differences were *p < 0.05 and **p < 0.01.

The efficacy of NHP in antioxidant activity was assessed using different *in vitro* cell-based assays. The scavenging of the DPPH radical is one of the important methods to investigate the scavenging effect of chemical constituents by reducing the oxidative stress *via* offering the hydrogen atoms and converting it into the non-toxic DPPH. After the incubation of NHP with DPPH, the change of color in the DPPH is the formal conformation of the scavenging practice. The DPPH-scavenging activity of NHP (50–500 µg/ml) increased in a dose-dependent manner (**Figure 7A**). Approximately 71% of DPPH was rummaged at a 500 µg/ml concentration of NHP and 400 µg/ml concentration (53%) was calculated as IC_50_. The results indicated that NHP could afford the hydrogen atom to scavenge the effective DPPH into a non-effective molecule. H_2_O_2_ is the key molecule to enhance the reactive oxygen species (ROS) level in the mitochondria, and scavenging the H_2_O_2_ is a substantial process of natural drugs in the field of redox biology. NHP breaks the H_2_O_2_ in a concentration (50–500 µg/ml)-dependent manner, and the results showed the H_2_O_2_ scavenging activities of NHP (**Figure 7B**). Almost 72% of the H_2_O_2_ molecule was broken at a higher concentration (500 µg/ml) of NHP, and the IC_50_ concentration was found to be 450 µg/ml, which was almost identical to the commercial antioxidant BHT. The conclusion of the findings is that NHP plays a key role in scavenging the ROS-inducing molecules.

Hydroxyl radicals are one of the dangerous as well as disease-causing molecules to the neighbor cells. In this study, NHP scavenged approximately 78% of the hydroxyl radical at 500 µg/ml and the IC_50_ of NHP was found to be 400 µg/ml (**Figure 7C**). The results were equated with the standard drug BHT, and the IC_50_ concentration of NHP was balanced with the BHT (350 µg/ml). Decreasing the oxidative efficiency of ferric chloride is another method to demonstrate the antioxidant potential of bioactive compounds. NHP decreased 75% of ferric chloride activity at 500 µg/ml concentration, while 51% of power was reduced by 400 µg/ml concentration that confirmed that NHP had a potential power-reducing ability but not as much as the commercial antioxidant BHT at 500 µg/ml (**Figure 7D**).

### 3.6 Cytotoxic Activity of N-(2-hydroxyphenyl)-2-phenazinamine

**Figure 8:**
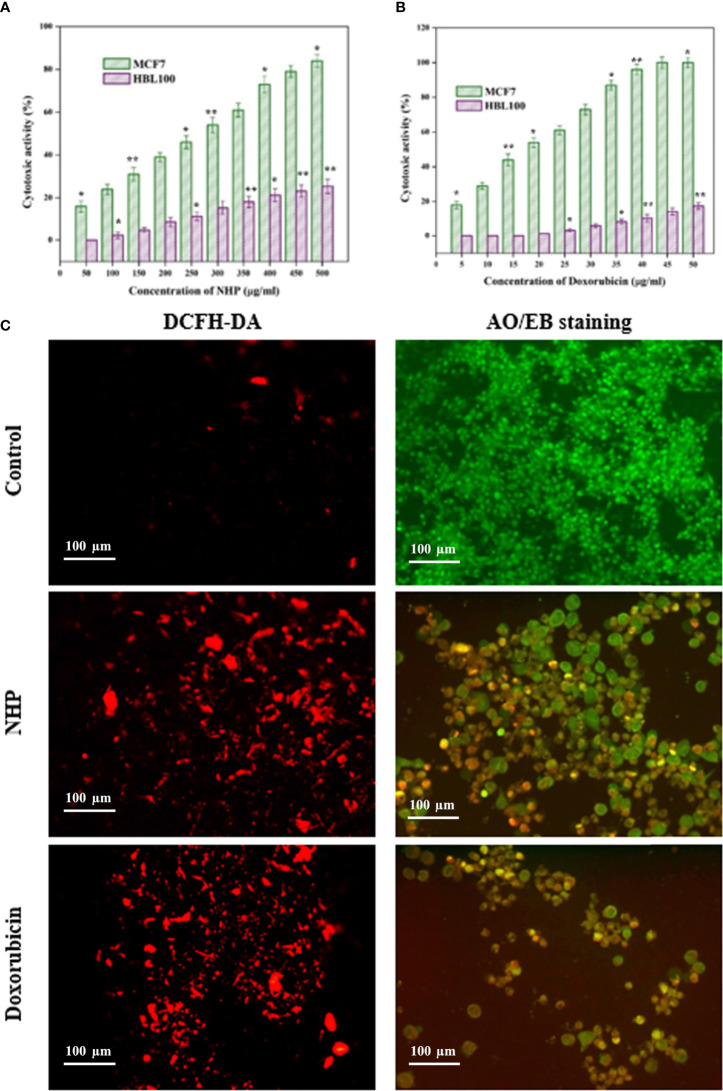
*In vitro* cytotoxic activity of NHP **(A)** and doxorubicin **(B)** against MCF7 breast cancer cells and HBL100 normal cells **(C)** showed the effect of NHP (300 µg/ml) on ROS generation and apoptosis in MCF7 cells using DCFH-DA and AO/EB staining, respectively. The results were taken in triplicates and expressed as mean ± standard deviation and the significant differences were *p < 0.05 and **p < 0.01.

The *in vitro* cytotoxicity activity against the MCF7 cell line was studied by the MTT assay at different concentrations of NHP and was compared with the standard doxorubicin. As shown in **Figure 8A**, NHP was able to reduce the viability of MCF7 cells in a dose-dependent manner, and the cytotoxic activity of NHP increases with increase in the concentration. The IC_50_ concentration of NHP was found to be 300 µg/ml, and the higher concentration of NHP (500 µg/ml) showed 84% of cytotoxic activity against MCF7 cells, while doxorubicin induced 50% of cytotoxicity at 20 µg/ml (**Figure 8B**). At the same time, the treatment of normal HBL100 cells with NHP (0–500 µg/ml) showed less cytotoxic activity, indicating that NHP had less toxicity.

### 3.7 Effect of N-(2-hydroxyphenyl)-2-phenazinamine on Reactive Oxygen SpeciesGeneration in MCF7 Cells

ROS-mediated oxidative stress regulates the various cancer signaling pathways and is a possible mediator of the apoptosis in the cancer cells. In order to elucidate the association between the cytotoxic activity and oxidative stress, the MCF7 cells were treated with NHP for 24 h and stained with DCFH-DA stain. The results showed that NHP increases the ROS generation in the mitochondria of MCF7 cells by increasing the fluorescence intensity in the treated MCF7 cells than in the control cells (**Figure 8C**).

### 3.8 Effect of N-(2-hydroxyphenyl)-2-phenazinamine on Apoptosis of MCF7 Cells

In order to reveal the relationship between oxidative stress and apoptosis, the MCF7 cells were treated with NHP for 24 h and stained with AO/EB dual stain. The results showed that the NHP-treated MCF7 cells showed the early and late stages of apoptotic cells by yellow green- and orange-colored cells, respectively (**Figure 8C**). The results indicate that the NHP derived from *N. exhalans* induces the apoptosis in MCF7 cells through the generation of oxidative stress in the mitochondria.

### 3.9 *In Vivo* Acute Toxicity

**Figure 9:**
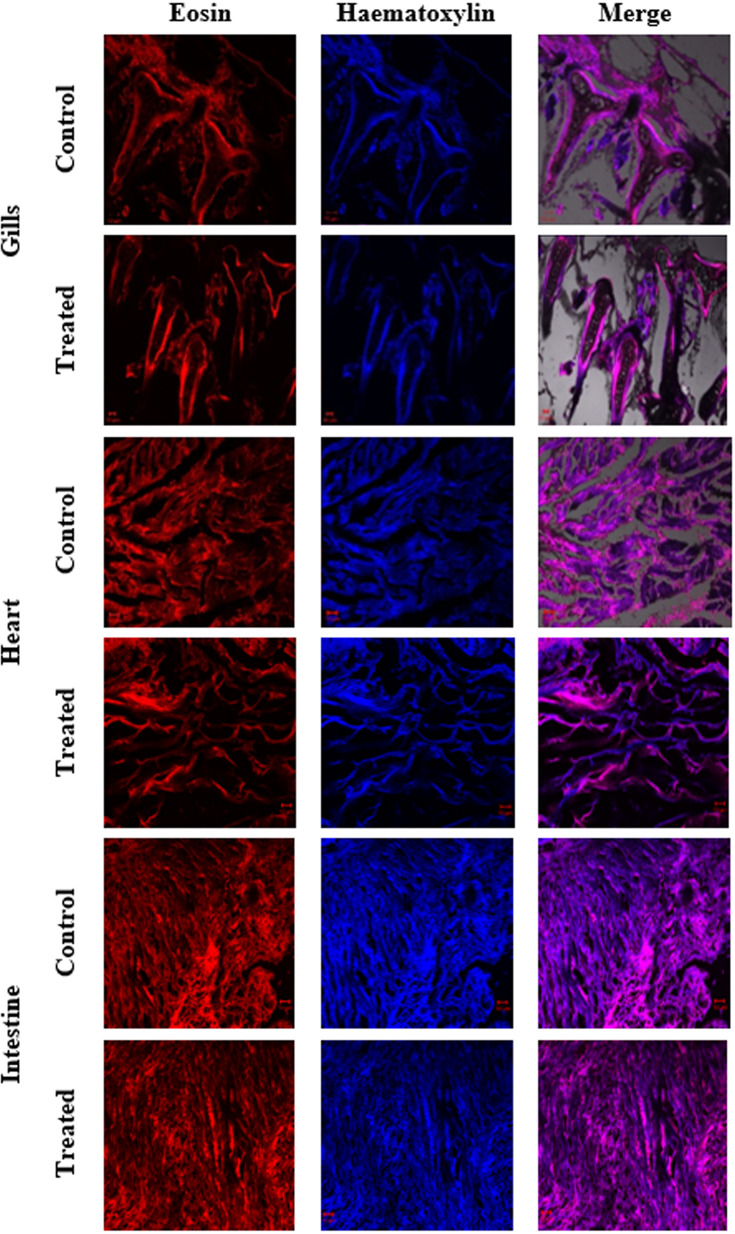
Histological changes in major organs (gills, heart, and intestine) of control and treated zebrafish *D. rerio* were evaluated after the 21-day exposure of NHP (500 µg/L).

**Figure 10:**
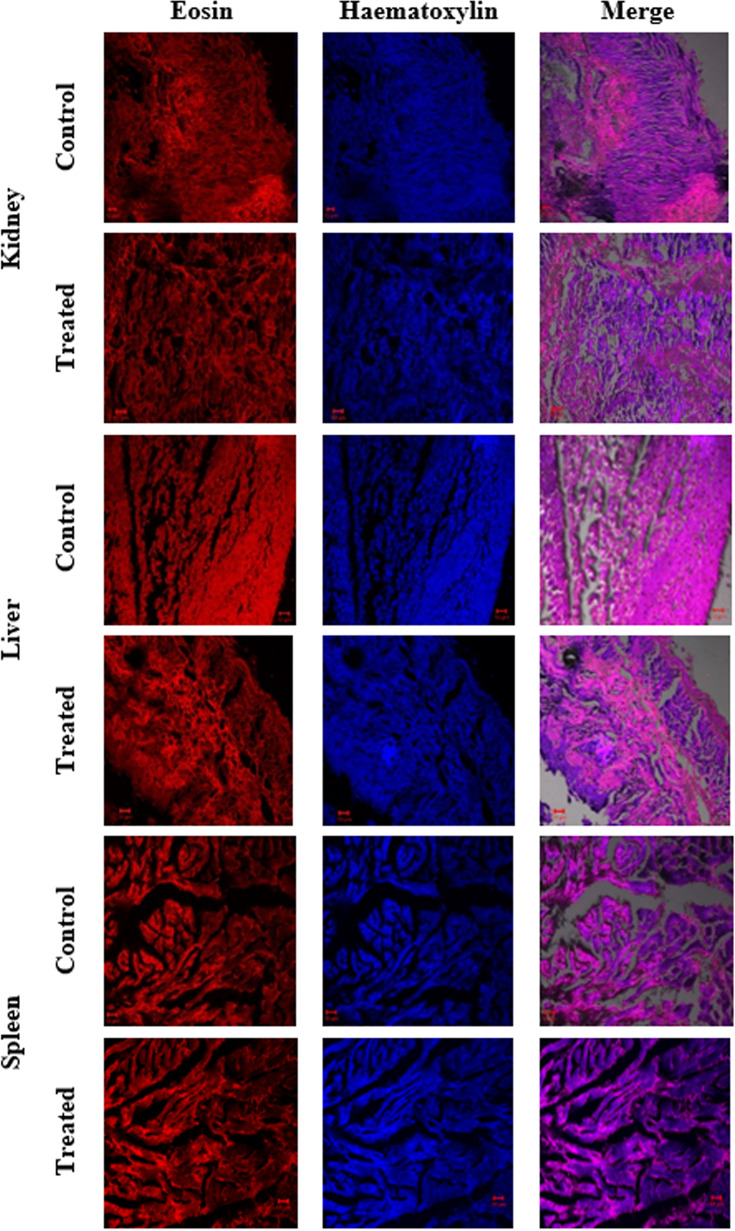
Histological changes in major organs (kidney, liver, and spleen) of control and treated zebrafish *D. rerio* were evaluated after the 21-day exposure of NHP (500 µg/L).

In the present study, the acute toxicity of NHP toward zebrafish *D. rerio* was evaluated by a 21-day treatment with NHP (500 µg/L). The results disclosed that only few deaths (>5%) were detected throughout the 21 days of treatment. Further, the histopathology of zebrafish organs such as gills, heart, intestine, kidney, liver, and spleen was done to study the effect of NHP. A histological study of gills revealed the distinctive morphological order of primary and secondary lamellae in the control and NHP exposure group of *D. rerio*. Additionally, NHP does not cause toxicity in the structure of heart after 21 days, in comparison with the control. The well-structured hepatocytes in liver, normal glomerular capillariae, mesengial cell hypertrophy, Bowman’s capsule layers in kidney, normal connective tissues, and lumen in intestine was detected in both control and NHP-exposed zebrafish (**Figures 9**, **10**).

## 4 Discussion

The prevalence of microorganisms producing secondary metabolites from marine environments may be associated with the self-protective or antagonistic roles of the organisms for maintaining their ecological niche in the environments (Singh et al., 2014). Further, the development of microorganisms in such aggressive environments makes way for an expectation that they may have metabolic activity against multidrug-resistant bacteria, which is the key for the development of antibiotics (Pham et al., 2019). Since the emergence of multidrug-resistant pathogens and the ongoing encounters for the treatment of infectious diseases, there is a high increasing demand for novel and effective antibiotics from actinomycetes (Chevrette et al., 2019). Hence, the present study was performed to search for novel antibiotics from marine actinomycetes *N. exhalans* isolated from the mucus of Scleractinia coral *A. formosa*.

Totally, 21 mucus-associated actinomycetes were isolated and the potential antimicrobial activity-possessing strain was selected for mass culture. Ethyl acetate was used for the crude extracts of *Streptomyces albogriseolus*, and the extract showed high antimicrobial activity against various bacterial and fungal pathogens (Sengupta et al., 2015). Likewise, in the present-study ethyl acetate was used for extraction of active compounds and evaluated for bacterial growth inhibitory activity. Previously, the ethyl acetate extract of actinomycetes derived from coral mucus showed good antimicrobial activity against coral pathogens (Li et al., 2014) and the corals afford livelihood for marine actinobacteria, which showed the genetic variability to produce a variety of potentially active compounds (Nithyanand et al., 2009).

Thin-layer chromatography was performed, and a total of six fractions were isolated and screened for antimicrobial activity. Among the six fractions, F4 has excellent bacterial growth inhibitory activity and was chosen for chemical constituent analysis. Similarly, six fractions were purified from marine actinomycete *Amycolatopsis alba*, and the one that showed the high antibacterial activity was selected for further characterization (Dasari et al., 2012). The partially purified crude extract using TLC showed significant activity against fish pathogens, and these results demonstrated that the *S. ruber* could be a novel antibiotic producer (Barakat and Beltagy, 2015).

Based on the characterization of F4, the purified compound was identified as N-(2-hydroxyphenyl)-2-phenazinamine. Our results were confirmed with previous results that showed that the FTIR spectrum of *S. fradiae* showed the presence of phenol group, alkene group, and nitrile group with aliphatics and primary amines (Prakash et al., 2015). Previously, the actinomycete strain *Nocardia dassonvillei* was able to produce phenazinamine with hydroxyl and aromatic groups in ^1^H NMR and carbonyl and aromatic carbons with methyl carbon signals in ^13^C NMR (Gao et al., 2012; Gao et al., 2013).

The isolated NHP from *N. exhalans* was investigated for antibiofilm activity against human clinical pathogens, and the results showed that NHP has exceptional antibiofilm activity in a concentration basis. Additionally, the CLSM analysis showed that NHP damages the architecture of the biofilm-forming clinical pathogens such as *E. coli*, *P. aeruginosa*, and *S. aureus* in comparison with the control. Previously, it has been reported that the bioactive compounds from coral-associated marine actinomycete *S. akiyoshienesis* inhibit the biofilm formation of clinical *Staphylococcal* biofilms (Bakkiyaraj and Karutha Pandian, 2010). Together, the NHP identified from the coral mucus-associated actinomycete showed biofilm inhibitory activity that was confirmed by the CLSM analysis.

Further, the antioxidant activity of NHP was evaluated and the results were equated with the standard antioxidant drug BHT, which designated that NHP had more or less comparable activity with BHT and discloses that NHP has the potential to be a scavenger. Previously, it has been reported that the sesquiterpenes derived from marine actinomycetes has potential to be a DPPH scavenger by donating an atom (Naine et al., 2015). ROS are a group of secondary products that comprise extremely oxidative stress-inducing molecules and the stable “diffusible” non-radical oxidants including H_2_O_2_ (Hayyan et al., 2016). Previously, it was reported that the marine actinomycetes derived from corals showed excellent antioxidant activity by scavenging the oxygen radical molecules and was used for the treatment of metabolic disorders (Poongodi et al., 2014; Sugiyama and Hirota, 2014). In this present study, the *in vitro* antioxidant studies confirmed that NHP could scavenge the cancer-causing molecules and act as a potential anticancer drug. Marine actinomycetes *S. variabilis* and *Streptomyces* sp. S2A showed dose-dependent *in vitro* antioxidant activity (Dholakiya et al., 2017; Siddharth and Vittal, 2018).

The intracellular antioxidant system manages the metabolic balance of ROS in the mitochondria of cancer cells; however, treatment by chemotherapy or drugs leads to the imbalance of ROS that increases the oxidative stress-mediated apoptosis (Ramalingam and Rajaram, 2021). Moreover, NHP inhibited the proliferation of MCF7 breast cancer cells in a dose-dependent manner and the results were compared with the standard anticancer agent doxorubicin. The cytotoxicity was also observed in human non-cancerous HBL100 cells treated with NHP. This emphasizes that NHP could be a potential anticancer agent since it has no toxicity against normal cells. Previously, 1-hydroxy-1-norresistomycin derived from coral mucus-associated actinomycetes showed potent anticancer activity against human lung cancer cells (A549) by inducing the p53-mediated intrinsic apoptosis signaling pathway (Ramalingam et al., 2018). Additionally, the treatment with NHP increased the ROS generation in MCF7 cells and induce the early and late apoptosis.

Histopathology was used to study the toxicological and morphological effects of drugs in the cell or tissue (van der Ven et al., 2003). In the present study, we investigated the histological changes in the 21-day exposure of NHP in zebrafish and the results showed that NHP did not induce any damage or structural modifications in the major organs of zebrafish. Previously, we reported that the 1-hydroxy-1-norresistomycin derived from marine actinomycetes *S. variabilis* induces less toxicity against *D. rerio* (Ramalingam and Rajaram, 2016). Together, these findings suggest that the NHP from *N. exhalans* does not cause any toxicity in zebrafish.

In conclusion, the coral mucus-associated actinomycete *N. exhalans* was used to produce a potential bioactive compound. Initially, the compound was isolated from the ethyl acetate extract and identified as N-(2-hydroxyphenyl)-2-phenazinamine using various spectroscopic analyses. As well, NHP showed good biofilm inhibitory activity against human clinical pathogens such as *E. coli*, *P. aeruginosa*, and *S. aureus*. Further, the *in vitro* antioxidant activity showed that NHP can scavenge the oxidative stress-generating molecules. The *in vitro* cytotoxic activity of NHP against breast cancer showed substantial activity, and less cytotoxic activity was also obtained against normal cells. The acute toxicity assay on *D. rerio* revealed that NHP has no toxicity, and the results were confirmed by the histopathological analysis of major organs in zebrafish. Together, these findings showed that NHP can act as potential antioxidant, since it has no toxicity and further studies will be carried out on the anticancer activity against human cancer.

## Data Availability Statement

The datasets presented in this study can be found in online repositories. The names of the repository/repositories and accession number(s) can be found in the article/**Supplementary Material**.

## Ethics Statement

Zebrafish (*Danio rerio*) was used as an experimental model organism and is not governed by any law. Therefore, the organisms were not placed for Institutional Animal Ethical Committee (IAEC) approval. All the experiments were performed for best practice according to Bharathidasan University IAEC guidelines. It is also informed that, as per the guidelines of the CPCSEA (Committee for the Purpose of Control and Supervision of Experiments on Animals), the use of this fish is not subject for approval by the IAEC. Furthermore, we did not conduct any experimentation with human subjects.

## Author Contributions

VR and RR – Designed the work. VR and GA – Methodology and toxicity analysis. VR and RR – Data acquiring and analysis. PP and BG – Funding acquisition. All authors contributed to the article and approved the submitted version.

## Conflict of Interest

The authors declare that the research was conducted in the absence of any commercial or financial relationships that could be construed as a potential conflict of interest.

The reviewer AK declared a shared affiliation with the authors RR and GA to the handling editor at the time of review.

## Publisher’s Note

All claims expressed in this article are solely those of the authors and do not necessarily represent those of their affiliated organizations, or those of the publisher, the editors and the reviewers. Any product that may be evaluated in this article, or claim that may be made by its manufacturer, is not guaranteed or endorsed by the publisher.
