# Supplementary material for: Structural Characterization, Antimicrobial, Antibiofilm, Antioxidant, Anticancer and Acute Toxicity Properties of N-(2-hydroxyphenyl)-2-phenazinamine From Nocardiopsis exhalans (KP149558)
Source: Front Cell Infect Microbiol. 2022 May 19;12:794338. doi: 10.3389/fcimb.2022.794338 (PMC9161293; doi:10.3389/fcimb.2022.794338)

**Supporting information**

Structural characterization, antimicrobial, antibiofilm, antioxidant, anticancer and acute toxicity properties of N-(2-hydroxyphenyl)-2-phenazinamine from *Nocardiopsis exhalans* (KP149558)

Vaikundamoorthy Ramalingam^a, b^*, Rajendran Rajaram^b,^ *, Govindaraju Archunan^c^, Parasuraman Padmanabhan^d,^ *, and Balázs Gulyás^e^

^a^Centre for Natural Products and Traditional Knowledge, Indian Institute of Chemical Technology, Hyderabad, Telangana, India.

**^b^**DNA Barcoding and Marine Genomics lab, Department of Marine Science, Bharathidasan University, Tiruchirappalli, India.

**^c^**Department of Animal Science, Bharathidasan University, Tiruchirappalli 620 024, Tamil Nadu, India

**^d^**Centre for Neuroimaging Research, Nanyang Technological University (NTU), Lee Kong Chian School of Medicine, 59, Nanyang Drive, Experimental Medicine Building, Singapore 636921.

**^e^**Lee Kong Chian School of Medicine, Nanyang Technological University (NTU), 59, Nanyang Drive, Experimental Medicine building, Singapore 636921

***Correspondence:** gene717ram@gmail.com; ppadmanabhan@ntu.edu.sg

**Table S1** shows the list of human clinical pathogens have used for the present study.

| **Pathogens** | **Name of the pathogen** |
| --- | --- |
| 1 | *Escherichia coli* (MH701895) |
| 2 | *Vibrio cholerae** |
| 3 | *Salmonella* sp*.** |
| 4 | *Klebsiella pneumoniae* (B938) |
| 5 | *Aeromonas* sp.* |
| 6 | *Proteus* sp*.** |
| 7 | *Corynebacterium* sp*.** |
| 8 | *Bacillus* sp*.* (MTCC 443) |
| 9 | *Staphylococcus aureus* (MTCC 96) |
| 10 | *Pseudomonas aeruginosa** |
| 11 | *Staphylococcus epidermis** |
| 12 | *Shigella sp.** |
| 13 | *Klebsiella sp.** |
| 14 | *Bacillus spp.** |
| 15 | *Pseudomonas sp.* (MTCC 424) |
| 16 | *Enterobacter sp.** |
| 17 | *Mycobacterium sp.** |
| 18 | *Staphylococcus sp.** |
| 19 | *Enterobacter (R)** |
| 20 | *Streptococcus sp.** |
| 21 | *Vibrio sp.** |

* - Pathogens procured from Government Hospital, Tiruchirappalli, Tamil Nadu, India. Rest of the pathogens were collected from Institute of Microbial Technology, CSIR, Chandigarh, India and the accession number were included.

**Table S2** shows the antimicrobial activity of 21 marine actinomycetes isolated from the mucus of Acropora formosa against human clinical pathogens using streak plate method

| **Strains** | **Human Pathogens** | | | | | | | | | | | | | | | | | | | | |
| --- | --- | --- | --- | --- | --- | --- | --- | --- | --- | --- | --- | --- | --- | --- | --- | --- | --- | --- | --- | --- | --- |
|  | 1 | 2 | 3 | 4 | 5 | 6 | 7 | 8 | 9 | 10 | 11 | 12 | 13 | 14 | 15 | 16 | 17 | 18 | 19 | 20 | 21 |
| **A.f – 1** | - | - | - | - | - | - | - | - | - | - | - | - | - | - | - | - | - | - | - | - | - |
| **A.f – 2** | 14 | 17 | 18 | 16 | 12 | 22 | 22 | 32 | 24 | 21 | 14 | 11 | 22 | 25 | 22 | 20 | 21 | 19 | 17 | 15 | 12 |
| **A.f - 3** | 11 | 7 | 16 | 17 | 15 | 19 | 21 | 16 | 25 | 15 | 13 | 12 | 18 | 14 | 21 | 12 | - | 28 | 18 | 21 | 17 |
| **A.f - 4** | 12 | 16 | 20 | 12 | 11 | 16 | 21 | 16 | 25 | 15 | 16 | 13 | 20 | 24 | 20 | 17 | - | 15 | 18 | 14 | 24 |
| **A.f - 5** | 10 | 11 | 7 | 14 | 15 | 17 | 22 | 24 | 17 | 13 | 14 | 10 | - | 15 | 19 | 19 | 21 | 26 | 16 | 15 | 12 |
| **A.f – 6** | 17 | 15 | 19 | 16 | 18 | 16 | 16 | 17 | 12 | 16 | 21 | 23 | 18 | 27 | 23 | 17 | 14 | 21 | 23 | 25 | 20 |
| **A.f - 7** | 14 | 11 | 23 | 19 | - | - | - | - | - | - | - | 17 | - | 16 | 14 | 19 | 20 | 25 | 19 | - | 12 |
| **A.f - 8** | 14 | 13 | 12 | 16 | 12 | 21 | 21 | 19 | 20 | 25 | 11 | 8 | 16 | 10 | 14 | - | 25 | 21 | 29 | 18 | 16 |
| **A.f - 9** | - | - | - | - | - | - | - | - | - | - | - | - | - | - | - | - | - | - | - | - | - |
| **A.f - 10** | 12 | 14 | 11 | 19 | 16 | 11 | 10 | 16 | 9 | 8 |  |  |  |  |  |  |  |  |  |  |  |
| **A.f - 11** | 11 | 14 | 13 | 12 | 14 | 13 | 15 | 10 | 21 | 9 | 17 | 16 | 15 | 11 | 12 | 18 | - | 21 | 14 | 20 | 10 |
| **A.f - 12** |  | 14 | 14 | 17 | 12 | 14 | 15 | 7 | - | 7 | - | 14 | 17 | 13 | 16 | 14 | 19 | 24 | 13 | 21 | 20 |
| **A.f - 13** | 12 | H | 18 | 12 | 11 | 19 | 12 | 17 | 18 | 14 | 11 | - | 10 | 12 | 11 | 20 | 19 | 29 | 21 | - | 29 |
| **A.f - 14** | - | - | - | - | - | - | - | - | - | - | - | - | - | - | - | - | - | - | - | - | - |
| **A.f - 15** | 13 | H | 18 | 24 | 23 | 16 | 18 | 14 | 12 | 21 | 12 | 16 | 10 | 21 | 22 | 10 | 26 | 14 | 21 | 31 | 21 |
| **A.f - 16** | 11 | H | 24 | 17 | - | - | - | 14 | - | 14 | 8 | - | 14 | 11 | - | 16 | - | - | 20 | - | 20 |
| **A.f - 17** | 12 | 16 | 18 | 24 | - | 20 | - | 15 | - | - | - | - | 17 | 15 | 13 | 16 | 29 | 21 | 29 | 26 | 20 |
| **A.f - 18** | - | - | - | - | - | - | - | - | - | - | - | - | - | - | - | - | - | - | - | - | - |
| **A.f - 19** |  |  |  |  |  |  |  |  |  |  | 7 | 21 | 11 | 11 | 8 | 21 | 14 | 28 | 30 | 20 | 22 |
| **A.f - 20** | - | - | - | - | - | - | - | - | - | - | - | - | - | - | - | - | - | - | - | - | - |
| **A.f - 21** |  |  |  |  |  |  |  |  |  |  | 10 | 13 | 11 | 14 | 16 | 14 | 26 | 13 | 30 | 12 | 18 |

**Fig. S1** Phylogenetic analysis of *Nocardiopsis exhalans*


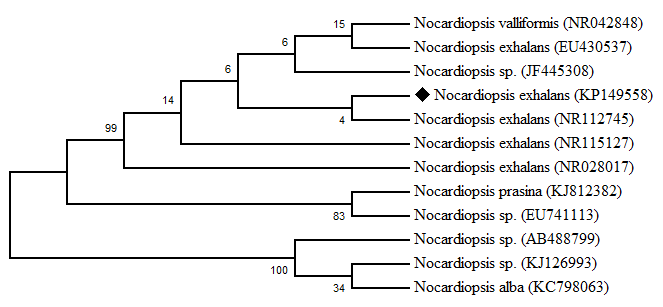


**Fig. S2** Mass data for the compound NHP


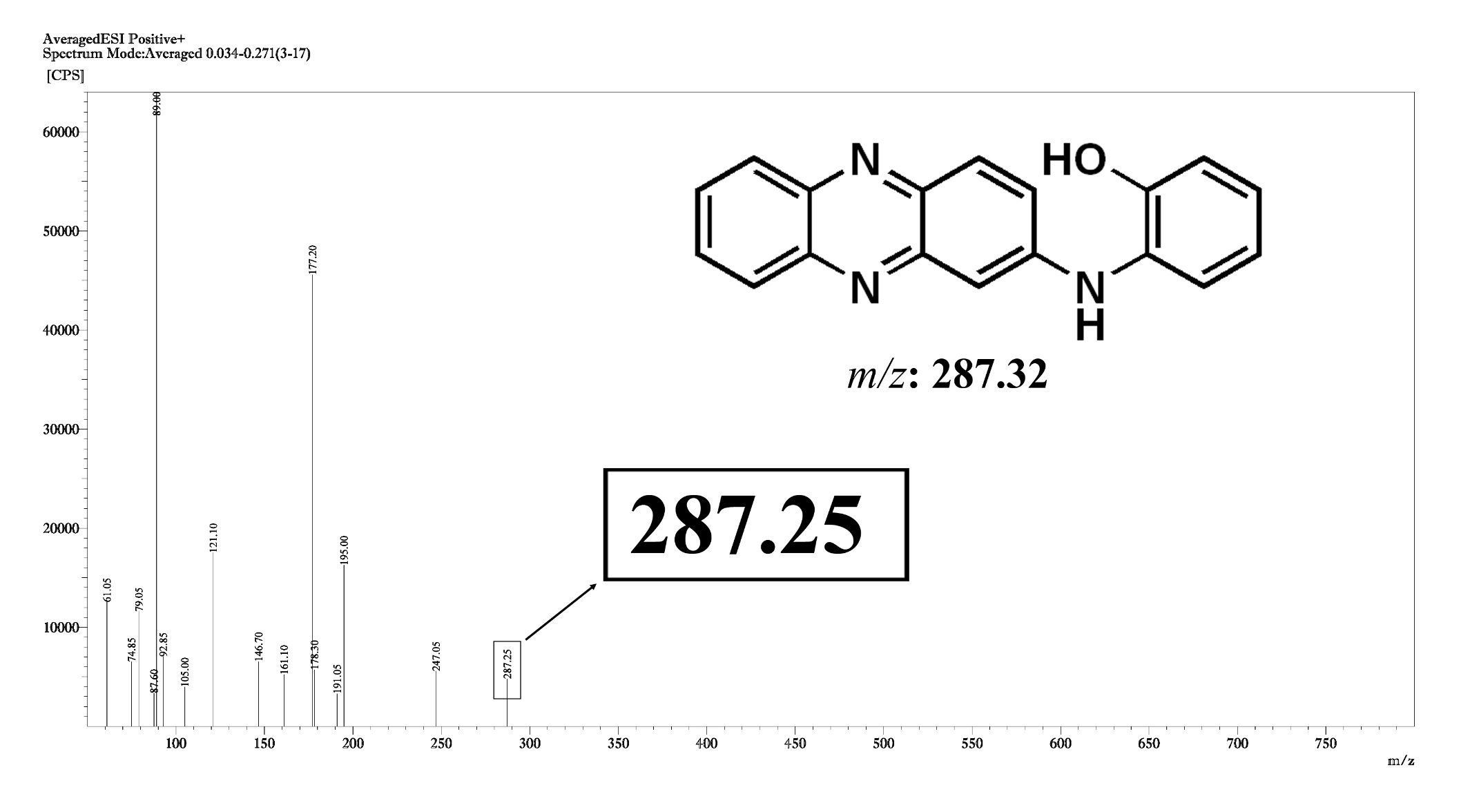

Supplement: Supplementary file 1 [file DataSheet_1.docx]
